# A Two-Step Lyssavirus Real-Time Polymerase Chain Reaction Using Degenerate Primers with Superior Sensitivity to the Fluorescent Antigen Test

**DOI:** 10.1155/2014/256175

**Published:** 2014-04-15

**Authors:** Vanessa Suin, Florence Nazé, Aurélie Francart, Sophie Lamoral, Stéphane De Craeye, Michael Kalai, Steven Van Gucht

**Affiliations:** ^1^National Reference Centre of Rabies, Viral Diseases, Communicable and Infectious Diseases, Scientific Institute of Public Health (WIV-ISP), Engeland Street 642, 1180 Brussels, Belgium; ^2^Toxoplasma Laboratory, Food-borne Pathogens, Communicable and Infectious Diseases, Scientific Institute of Public Health (WIV-ISP), Engeland Street 642, 1180 Brussels, Belgium

## Abstract

A generic two-step lyssavirus real-time reverse transcriptase polymerase chain reaction (qRT-PCR), based on a nested PCR strategy, was validated for the detection of different lyssavirus species. Primers with 17 to 30% of degenerate bases were used in both consecutive steps. The assay could accurately detect RABV, LBV, MOKV, DUVV, EBLV-1, EBLV-2, and ABLV. *In silico* sequence alignment showed a functional match with the remaining lyssavirus species. The diagnostic specificity was 100% and the sensitivity proved to be superior to that of the fluorescent antigen test. The limit of detection was ≤1 50% tissue culture infectious dose. The related vesicular stomatitis virus was not recognized, confirming the selectivity for lyssaviruses. The assay was applied to follow the evolution of rabies virus infection in the brain of mice from 0 to 10 days after intranasal inoculation. The obtained RNA curve corresponded well with the curves obtained by a one-step monospecific RABV-qRT-PCR, the fluorescent antigen test, and virus titration. Despite the presence of degenerate bases, the assay proved to be highly sensitive, specific, and reproducible.

## 1. Introduction

Rabies is a fatal viral encephalitis that results from infection with negative strand RNA-viruses belonging to the genus* Lyssavirus*, family Rhabdoviridae, order Mononegavirales. So far, 12 species have been classified in the genus* Lyssavirus*. Traditionally, these include Rabies virus (RABV), Lagos bat virus (LBV), Mokola virus (MOKV), Duvenhage virus (DUVV), European bat lyssaviruses-1 and -2 (EBLV-1 and EBLV-2), and Australian bat lyssavirus (ABLV). More recently, Aravan virus (ARAV), Khujand virus (KHUV) [1], Irkut virus (IRKV) [1], Shimoni bat virus (SHIBV), and West Caucasian bat virus (WCBV) [1] were also added. Ikoma virus (IKOV) [2] and Bokeloh bat lyssavirus (BBLV) [3, 4] await classification in the genus. The genus is subdivided into phylogroups 1 and 2 [5, 6]. Phylogroup 1 includes RABV, DUVV, EBLV-1, EBLV-2, ABLV, ARAV, KHUV, and IRKV. Phylogroup 2 includes LBV, MOKV, and SHIBV. WCBV and IKOV do not cross-react serologically with any of the two phylogroups.

The classic rabies virus (RABV) has a worldwide distribution and uses carnivores and bats as main reservoir. The other lyssavirus species are mainly maintained in bats and have a more restricted distribution: DUVV, LBV, MOKV, SHIBV, and IKOV have been detected exclusively in Africa, EBLV-1, -2 and BBLV in Europe, ABLV in Australia, and ARAV, KHUV, IRKV, and WCBV in Asia. It is assumed that most lyssaviruses can cause the rabies syndrome in humans and other mammals [6–8]. In Western Europe, most cases of rabies in humans or pets are imported and may be caused by any species within the Genus* Lyssavirus* [9, 10]. For example, in 2007, in The Netherlands, a patient died from infection with the rare DUVV upon return from Kenya [11]. Moreover, locally acquired infections in humans and cats with EBLV-1 or EBLV-2 are also possible on the European territory [12]. A diagnostic assay that can rapidly detect all species is therefore highly recommended.

Currently, the gold standard methods for the diagnosis of rabies recommended by the World Health Organisation (WHO) are the fluorescent antibody test (FAT), the rabies tissue culture infection test (RTCIT), and the mouse inoculation test (MIT) [13–18]. The FAT is convenient for* postmortem* examination and detects the presence of viral nucleocapsid antigens in the brain or spinal cord tissue by staining with specific fluorescent antibodies [14]. For* antemortem* diagnosis of rabies, the presence of viral antigen can be detected with the FAT in tissue sections of skin biopsies, typically in the nerve endings surrounding the hair follicles. The viral antigens are however often only detectable at the end of the disease or cannot be detected at all by this method [17]. Repeated sampling is necessary to improve the diagnostic sensitivity. This is not practical for skin biopsies [17] but easier for body fluids, such as saliva, urine, or cerebrospinal fluid. The sensitivity of the FAT method is considered high for RABV but may be lower for other lyssavirus species [19–21].

RTCIT and MIT are based on the isolation and propagation of virus, respectively, in cell culture or in mice [22]. Isolation of the virus from body fluids requires the presence of infectious virus in the sample and the absence of viral inhibitors or antibodies and is time consuming. Antirabies virus antibodies acquired either by natural seroconversion, by treatment with immunoglobulins, or after a postexposure vaccination can interfere with the virus isolation from clinical samples, possibly yielding false negative results in the RTCIT and MIT. In our experience, MIT and RTCIT are very specific methods but are restricted to samples containing live and uninhibited virus. Furthermore, neither the FAT, RTCIT, or MIT can directly distinguish between different lyssavirus species. Seroconversion during the course of disease is highly indicative for rabies, but patients often receive treatment with antirabies immunoglobulins and vaccine, compromising the interpretation of serology. Molecular techniques have recently been developed for rabies virus diagnosis. Viral RNA can be extracted from several matrices, such as saliva, urine, cerebrospinal fluid (CSF), or skin tissue, and do not require the presence of live virus. RT-PCR can therefore be used under a broad range of conditions. RT-PCR has been shown to detect RNA in decomposed samples [23] or after long-term storage [24], giving a better chance of successful diagnosis than RTCIT [25]. Also, the qRT-PCR method can allow to distinguish different lyssavirus species.

Here, we aimed to develop and validate a nested two-step generic lyssavirus real-time RT-PCR (qRT-PCR) protocol, combining the use of degenerate primers with real-time PCR detection. A two-step approach was chosen to maximize the sensitivity of the assay. Degenerate bases were included in the primers at key positions to account for the variability in the sequence of the different lyssavirus species. During the first amplification round (PCR1), the primers amplified a 343 bp fragment of the nucleoprotein N gene, whereas in the following real-time PCR a 158 bp fragment was amplified and detected using SYBR Green. The overall sensitivity, specificity, selectivity, and reproducibility of the assay were assessed by comparison with FAT. More specifically for RABV detection, the performance of the generic lyssavirus qRT-PCR was compared with a RABV-specific qRT-PCR protocol, using specific primers without degenerate bases. A large set of samples obtained from humans, naturally and experimentally infected wild and domestic animals were included to validate the assay.

## 2. Materials and Methods

### 2.1. Samples

#### 2.1.1. Negative Samples

Brain, serum, and cerebrospinal fluid (CSF) samples from different species (bat, red fox, dog, cat, mouse, and human) were used as negative controls (Table 1). All samples tested negative for rabies in the FAT and/or RTCIT gold standard methods. To further test for specificity, a virus suspension of vesicular stomatitis virus (VSV) (genus* Vesiculovirus*) was also tested to demonstrate the selectivity of the primers. VSV belongs to another genus within the family Rhabdoviridae and shares biological and genetic features with the lyssaviruses.

#### 2.1.2. Positive Samples

To test the performance of the generic lyssavirus qRT-PCR, samples spiked with different lyssaviruses were used (Table 2). Challenge Virus Standard-11 (CVS-11), a virulent neurotropic lyssavirus, was obtained from the American Type Culture Collection (ATCC: reference VR959). CVS-11 was grown in baby hamster kidney (BHK)-21 cells, as described previously [29].

Experimentally infected brain samples were prepared as described next. Female Swiss outbred mice (Harlan, The Netherlands) were inoculated intranasally at the age of 6 to 8 weeks, according to Rosseels et al. [29]. The experimental procedure was approved by the Local Ethical Committee of the WIV-ISP (advice nr. 060217-03). Sixty mice were each inoculated with 3 × 10^2^ 50% TCID_50_ of CVS-11 and euthanized by cervical dislocation from 0 to 10 days after inoculation (DPI). Five control mice received only phosphate-buffered saline (PBS). The evolution of the load of infectious virus, viral antigen, and viral RNA was followed for 10 days. The brain was cut in half and the inner part of the left half was pressed on a glass slide for FAT analysis. Both brain halves were then homogenized in 1 mL of PBS with a Potter homogenator. The homogenate underwent 3 consecutive freeze-thaw cycles at −80°C and was centrifuged at 20000 g for 20 min at 4°C. The supernatant was collected for virus titration (300 *μ*L), RABV-specific qRT-PCR, and generic lyssavirus qRT-PCR (2 × 85 *μ*L). Virus titration was performed according to the instructions of the Manual of Diagnostic Tests and Vaccines for Terrestrial Animals (Office International des Epizooties, 2013). Serial five-fold dilutions were added in triplicate to BHK-21 cells. After a 2-day incubation period, the cells were stained with fluorescein isothiocyanate (FITC)-coupled antinucleocapsid rabbit antibodies (Bio-Rad Laboratories, Hercules, USA) and the number of infected foci was counted. The titre of infectious virus was expressed in TCID_50_/mL.

### 2.2. Fluorescent Antibody Test (FAT)

The FAT was performed according to the instructions of the Manual of Diagnostic Tests and Vaccines for Terrestrial Animals (Office International des Epizooties, 2012). Briefly, the tissue slides were fixed in 75% acetone for 10 min at −20°C and stained with FITC-labelled antinucleocapsid rabbit antibodies (Bio-Rad Laboratories, Hercules, USA) for 30 min at 37°C. The slides were examined with a Nikon Diaphot 200 fluorescence microscope connected to a Moticam 2500 camera (Hong-Kong, China) at a magnification of 100x.

### 2.3. Generic Lyssavirus qRT-PCR

#### 2.3.1. RNA Extraction

Total RNA from the various samples was extracted using the Qiagen RNeasy Mini kit (for brain and cell culture medium) or the Qiagen QIAamp viral RNA Mini kit (for serum and CSF) (Qiagen, Hilden, Germany). Brain samples (400 mg) were first homogenised in 1 mL of PBS with a Potter homogenator. Eighty-five microliter of the brain homogenate was mixed with 265 µL of lysis buffer (RLT) and used for RNA extraction. From here on, the instructions from the RNeasy Mini kit were followed. For RNA extractions from infected cell culture supernatants, a volume of 150 µL was homogenised in 200 µL of RLT buffer, as starting material for the RNA extraction. Starting from serum or CSF, a volume of 140 µL was used for RNA extraction with the Qiagen QIAamp viral RNA Mini kit following the manufacturer's instructions.

#### 2.3.2. Reverse Transcription

The RNA extracts were quantified with a NanoVue spectrophotometer (GE healthcare, Bucks, United Kingdom). The reverse transcription reaction was performed using the qScript cDNA SuperMix (Quanta BioSciences, Gaithersburg, USA). Briefly, 18 µL containing 100 ng of RNA template in RNAse-free water and 2 µL of the qScript supermix were mixed and incubated for 5 min at 25°C followed by 30 min at 42°C and 5 min at 85°C.

#### 2.3.3. Design of the Oligonucleotide Primers

The nucleoprotein N gene was used as target for the qRT-PCR. The primers were synthesized by Eurogentec (Seraing, Belgium). The lyssavirus primers were chosen based on multiple alignments of the N gene sequence from RABV, LBV, MOKV, DUVV, EBLV-1, EBLV-2, ABLV, WCBV, KHUV, and IRKV. Complete sequences of the nucleoprotein gene of each lyssavirus species were obtained from genomic databases and aligned by using the MEGA5 and the CLC Sequence Viewer software. The accession numbers of these viruses are shown in Table 3(a). External and nested primer sequences were chosen from regions conserved in all lyssavirus species. The RAB PCR1 F, RAB PCR1 R, and RAB qPCR F primers were designed in-house (Table 3(b)). The GRAB2R primer was described earlier by Vázquez-Morón et al. [30]. Degenerate bases were included in the primers to account for the variability in the sequences of different lyssavirus species. A first amplification round (PCR1) was performed using the RAB PCR1 F and RAB PCR1 R primers, which amplify a fragment of 343 bp of the N gene. 5 µL of the PCR product was diluted 10x and then used in a real-time PCR with the RAB qRT-PCR F and GRAB2R primers, amplifying a 158 bp fragment within the first PCR amplicon. The RABV monospecific primers (RAB CVS-11 F and RAB CVS-11 R) were designed to specifically target the nucleoprotein N gene of CVS-11 (accession number GU992321). They contain no degenerate bases (Table 3(b)). The primers VETINHF2 and VETINHR1 were designed in-house to amplify a conserved sequence of the r18S ribosomal RNA gene and were usedto check for PCR inhibition and RNA quality [31, 32]. They can be used for samples from multiple animal species (Table 3(b)).

#### 2.3.4. Nested qPCR

The protocol consisted of a nested PCR strategy with two amplification steps. In the first amplification round (PCR1), 5 *μ*L of cDNA was mixed with a PCR master mix containing 10 *μ*L of 5x reaction buffer, 1.5 mM of MgCl_2_, 0.2 U Taq polymerase (Promega, Madison, USA), 0.5 *μ*M of the primers RAB PCR1 F and RAB PCR1 R, 0.2 mM of dNTP mix (Roche, Basel, Switzerland),and nuclease-free water to obtain a final volume of 50 µL. The amplification was performed on an iCycler (Biorad, Hemel Hempstead, United Kingdom) according to the following program: 1 min at 95°C for initial denaturation, followed by 25 cycles of 20 s at 95°C (denaturation) and 40 s at 60°C (annealing and extension), and a final step of 10 min at 72°C. The second amplification consisted of a real-time PCR performed on a CFX96 real-time PCR system (Biorad, Hemel Hempstead, United Kingdom). The reaction mix consisted of 12.5 µL of 2x SyberGreen Master Mix (Quanta BioSciences, Gaithersburg, USA), 5 µL of 10x diluted PCR1 product and 0.8 µM of each primer (RAB qRT-PCR F and GRAB2R), and nuclease-free water to obtain a final volume of 25 µL. All samples were analyzed in duplicate. The program consisted of 2 min at 95°C for Taq activation and initial denaturation, followed by 45 cycles of 20 s at 95°C and 30 s at 61°C. To check for the presence of primer dimers and nonspecific amplicons, a melting curve analysis was performed after each run (Bio-Rad CFX manager 2.1 software).

A total of 45 cycles were ran in the qPCR. The interpretation of the qPCR results was done as follows: a sample with a Cq value ≤40 was considered positive and a Cq value >40 or an undetectable Cq was considered negative. Samples with a Cq value between 40 and 45 were always retested. The melting curve was analysed to check the specificity of the amplification.

#### 2.3.5. Sequencing Species Identification

The obtained amplicons were purified using the GFX PCR DNA kit (GE Healthcare, PQ, USA) and quantified with the NanoVue spectrophotometer (GE Healthcare, PQ, USA). The sequencing reactions were performed using the RAB qRT-PCR F primer and the big dye terminator v.3.1 cycle sequencing kit (Applied Biosystem, CA, USA), according to the manufacturer's instructions. As starting material, 1–3 ng of the purified PCR fragment was used. The cycle PCR reactions were performed on an iCycler from Biorad, programmed as follows: a first denaturation step of 1 min at 96°C and 25 cycles consisting of 10 s at 96°C and 4 min at 60°C. Final products were purified by precipitation by adding 5 µL of 125 mM EDTA/sodium acetate 3 M solution and 60 µL of ethanol 100%. The sequencing reactions were analysed on an ABI Prism 3130 Genetic Analyzer (Applied Biosystem, California, USA). The obtained sequences were matched with the sequences stored in the National Center for Biotechnology Information (NCBI) database, using the Basic Local Alignment Search Tool (BLAST).

### 2.4. RABV-Specific qRT-PCR

The RABV-specific qRT-PCR consisted of a single amplification step and was performed on a CFX96 real-time system from Biorad (Hemel Hempstead, United Kingdom). Each 25 *μ*L reaction mixture consisted of 12.5 µL of 2x Sybr Green Master Mix (Quanta BioSciences, Gaithersburg, USA), 5 *μ*L of diluted cDNA, and 0.4 *μ*M of each primer. All samples were analyzed in duplicate. The amplification program consisted of 2 min initial denaturation at 95°C, followed by 45 cycles of 20 s at 95°C and 30 s of annealing and extension at 62°C. A melting curve analysis was performed in order to verify the absence of primer dimers and the specificity of the obtained amplicons after each run (Bio-Rad CFX manager 2.1 software). The correlation between Cq values of both qRT-PCR methods was calculated with the Pearson's correlation test (GraphPad Prism 6 software).

## 3. Results

### 3.1. Diagnostic Sensitivity 

#### 3.1.1. Spectrum of Lyssavirus Detection

**Figure 1:**
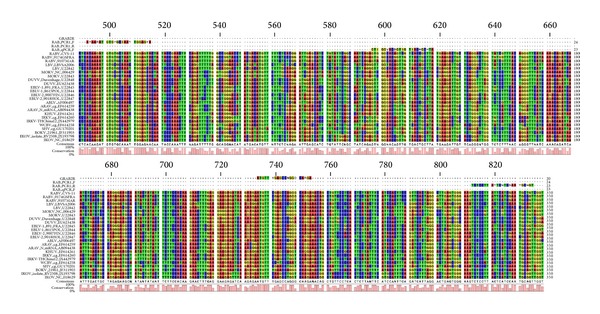
Sequence alignment of the degenerate primers with the targeted region of the nucleoprotein gene of 24 isolates of 14 different lyssavirus species.

All positive samples (*n* = 69) that contained either one of 7 different lyssavirus species (Table 2) were tested positive with the lyssavirus qRT-PCR, yielding a sensitivity of 100% for the assay. Some of the rare species (WCBV, IRKV, KHUV, IKOV, SHIBV, and BBLV) were not tested, but* in silico* alignment of the targeted region in the nucleoprotein gene of these species demonstrated a sufficient match with the degenerate primers to allow amplification (Figure 1). No primer dimers were detected. The obtained amplicons were also checked by agarose gel analysis and had the expected size of 158 bp (data not shown).

To demonstrate the superior sensitivity of the two-step approach, the lyssavirus qRT-PCR was compared with a previously published one-step lyssavirus qRT-PCR [33]. Serial dilutions of 2 virus-positive samples from the 2013 annual international proficiency test from ANSES (Nancy, France) were compared. The samples contained a RABV isolate from a dog (*Canis lupus familiaris*), or an EBLV-1 isolate from a bat (*Eptesicus serotinus*). The virus-positive samples were diluted ten-fold and tested by both protocols. The obtained results are presented in Table 4. The two-step approach was 10 times more sensitive than the one-step approach [33].

#### 3.1.2. Comparison of the Course of an Experimental RABV Virus Infection in Mice Determined by Generic Lyssavirus qRT-PCR, RABV Monospecific qRT-PCR, and FAT

**Figure 2:**
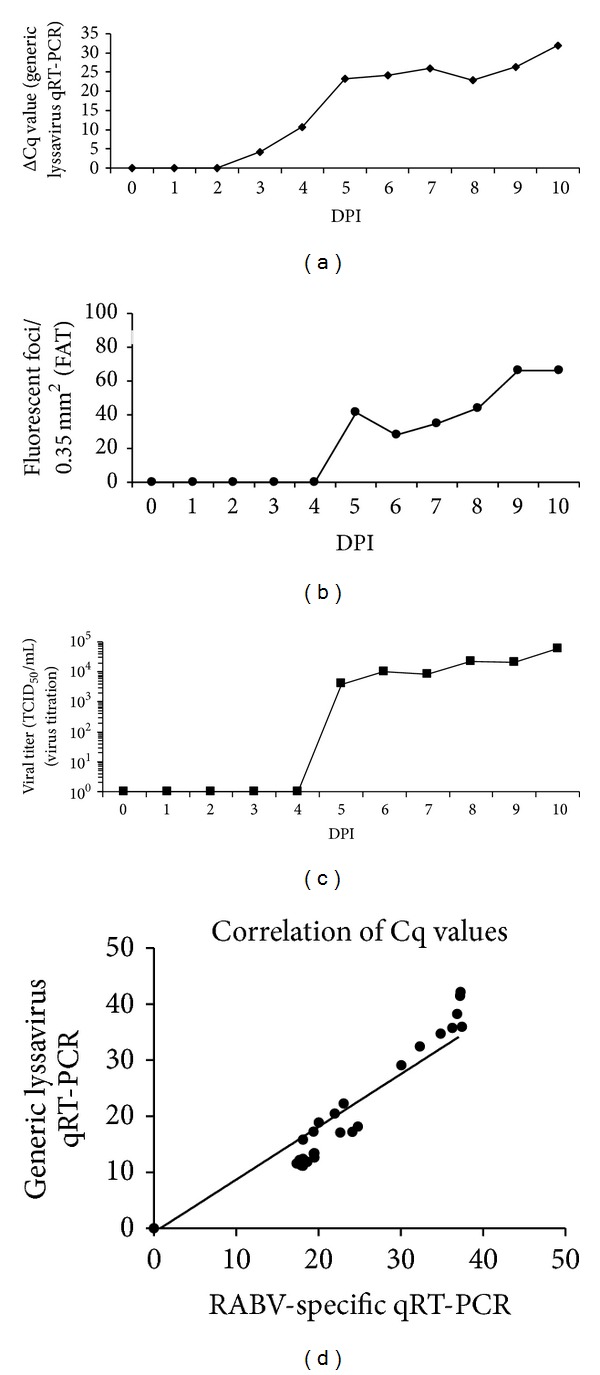
Kinetic profile of RABV infection in mice determined by generic lyssavirus qRT-PCR, FAT and virus titration. Mice were infected with 3 × 10^2^ TCID_50_ of RABV (CVS-11) by intranasal inoculation and sacrificed 0 to 10 days later. The brain was collected for analysis. The course of (a) viral RNA by generic lyssavirus qRT-PCR, (b) load of viral antigen (FAT), (c) infectious virus (RTCIT), and (d) correlation of Cq values between the generic lyssavirus and RABV-specific qRT-PCR are presented. The correlation between the Cq values obtained by both qRT-PCR methods was excellent (Pearson's correlation coefficient *r* = 0.9773, *P* < 0.0001).

Mice were inoculated intranasally with rabies virus and brain tissue was sampled at different DPI for analysis. qRT-PCR results were expressed in delta Cq values, which were calculated as follows: 45 − Cq_sample_ (45 = the total number of cycles run in the qPCR). Viral RNA was first detected at 3 DPI (mean delta Cq of 4.2), which was followed by an increase of the viral RNA load at 5 DPI (mean delta Cq of 23.2 at 5 DPI) (Figure 2(a)). The viral load reached a plateau from 7 DPI onwards (mean delta Cq of 26). Starting from 5 DPI, viral antigens could also be detected with the FAT and small amounts of infectious virus could be isolated from the brain (Figure 2(b)). At 5 DPI, the virus titer ranged from 10^2^ to 10^4^ TCID_50_/mL. The titer increased during the following days to reach a plateau, ranging between 10^4^ and 10^6^ TCID_50_/mL at 8 DPI (Figure 2(c)). The Cq values obtained with the one-step monospecific qRT-PCR correlated well with the values obtained with the two-step lyssavirus qRT-PCR (Pearson value *r* = 0.9773; *P* < 0.001, GraphPad Prism 6) (Figure 2(d)). The generic lyssavirus qRT-PCR proved to be more sensitive than the FAT. Eleven mice that tested positive with the generic lyssavirus qRT-PCR at 3 and 4 DPI, tested negative by the FAT. Uninfected control mice tested negative with both qRT-PCR methods and the FAT.

### 3.2. Selectivity

**Figure 3:**
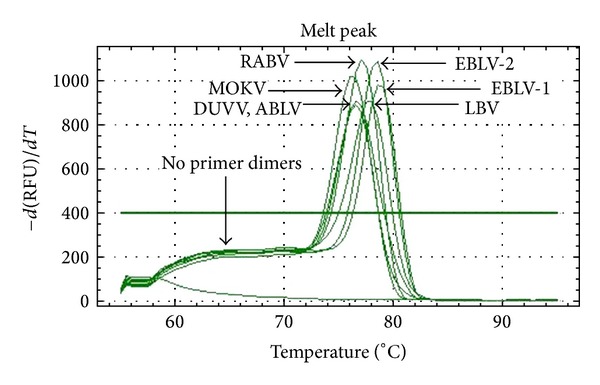
Melting peaks obtained for 7 lyssavirus species. The melting temperatures for RABV, MOKV, LBV, DUVV, EBLV-1, -2, and ABLV were, respectively, 77°C, 77.5°C, 76°C, 76.5°C, 78.5°C, 78°C, and 76.5°C. No primer dimers were observed.

For all samples, the sequence of the obtained amplicon could be assigned unequivocally to the correct species by comparing the nucleotide homology in the NCBI database (BLAST, highly similar sequences). Homology was in the range of 95–100% for RABV, MOKV, EBLV-1, EBLV-2, and ABLV and in the range of 85–87% for LBV and DUVV. Moreover, melting temperature profiles of the respective amplicons differed for the different species and no primer dimers were observed in any PCR run (Figure 3). The vesicular stomatitis virus (VSV) tested negative (undetectable Cq) with the generic lyssavirus qRT-PCR.

### 3.3. Limit of Detection

**Figure 4:**
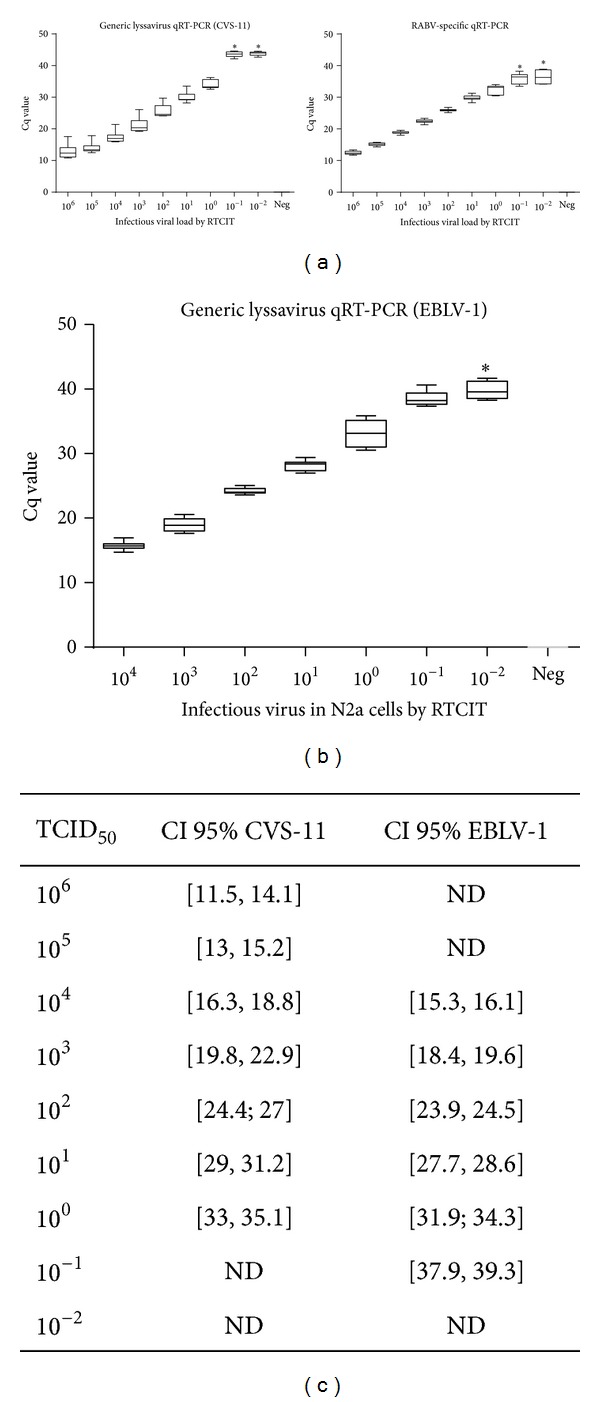
Analytical sensitivity of the monospecific RABV and the generic lyssavirus qRT-PCR for RABV (CVS-11) (a) and EBLV-1 (b). Six independent runs with each time two repeats were performed per virus dilution (10-fold serial dilution). There was an excellent linear regression between the load of infectious virus, determined by virus titration, and the Cq value for RABV and EBLV-1 (regression coefficient of 0.965 and 0.989, resp.). For both qRT-PCR methods, the limit of detection of RABV and EBLV-1 was ≤ 100 TCID_50_. The Cq remained undetectable in the negative control samples.*Mean and standard deviation are calculated based on the runs/repeats with a positive signal (Cq ≤ 40). ND = not determined.

The limit of detection was determined by analysing 10-fold serial dilutions of a suspension of RABV (CVS-11) and EBLV-1. RABV and EBLV-1 suspensions were produced in BHK-21 and neuroblastoma N2a cells, respectively. The titer of infectious virus was determined in cell culture. The virus was diluted from 10^6^ to 10^−2^ TCID_50_ for RABV and from 10^4^ to 10^−2^ TCID_50_ for EBLV-1. For RABV, the viral RNA load was determined by both the generic and the RABV-specific qRT-PCR, while for EBLV-1, only the generic lyssavirus qRT-PCR was used. The obtained Cq values correlated well with the logarithm of the corresponding TCID_50_ values using a linear fit model (GraphPad Prism 6) (Figure 4(a)).

The limit of detection for RABV was 10^0^ TCID_50_ for both qRT-PCR methods, with 100% positive replicates (6 different runs in duplicate). At the 10^−1^ TCID_50_ and 10^−2^ TCID_50_ dilutions, all replicates were negative (Cq value > 40 or undetectable) with the generic lyssavirus qRT-PCR. For the RABV monospecific qRT-PCR, 8 replicates were positive and 4 replicates were negative at the 10^−1^ TCID_50_ dilution. At the 10^−2^ TCID_50_ dilution, 4 replicates were positive and 8 replicates were negative with the RABV-specific qRT-PCR (Figure 4(a)). Negative control samples (viral RNA substituted by water) gave no signal with both qRT-PCR methods. The detection limit of EBLV-1 was determined at 10^−1^ TCID_50_ for the generic lyssavirus qRT-PCR. At 10^−1^ TCID_50_, all replicates yielded positive results. At 10^−2^ TCID_50_, 5 replicates yielded positive and 7 replicates yielded negative results (Cq value > 40 or undetectable) (Figure 4(b)).

The two-step protocol was also compared with a one-step qPCR round based on the same PCR1 or qPCR primers, using samples from the 2011 and 2013 annual ring test from ANSES (Nancy, France). A sample containing an ABLV isolate from an Australian bat (*Pteropus Alecto*) was diluted 1/10, 1/100, 1/500, and 1/1000 and a sample containing a RABV isolate from a European red fox (*Vulpes vulpes*) was diluted 1/8, 1/100, and 1/500. All dilutions tested positive with the two-step protocol. Depending on the dilution, Cq values ranged from 16 to 24. In contrast, the one-step protocol gave either no signal or high Cq values, ranging between 31 and 44 (data not shown). This underlines the need for the two amplification rounds to obtain acceptable sensitivity.

### 3.4. Diagnostic Specificity

The diagnostic specificity was 100%. A total of 150 brains of rabies virus-free mammals, belonging to 5 different species, tested negative by FAT and generic lyssavirus qRT-PCR (Table 1). Ten human cerebrospinal fluid samples, 6 saliva samples, and 1 skin biopsy obtained from rabies-free patients with neurological symptoms tested negative by qRT-PCR, yielding a diagnostic specificity of 100%. The qRT-PCR always yielded negative results upon substitution of the RNA by water in the different steps of the protocol (RNA extraction, reverse transcription, and two-step qPCR).

### 3.5. Repeatability (Intra-Assay Variation)

To assess the intra-assay variation, 3 samples (CVS-11, EBLV1, and water as a negative control) were tested in 10 replicates. The variation coefficient (Pearson* r*-GraphPad Prism 6) was 1.7% for CVS-11 (at a mean Cq of 21.5) and 0.89% for EBLV-1 (at a mean Cq of 24.3). The negative control remained undetectable in all replicates.

### 3.6. Reproducibility (Inter-Assay Variation)

The inter-assay precision was assessed by testing 3 samples (CVS-11, EBLV-1, and water as a negative control) in 6 independent runs (Figure 4). The virus suspensions were diluted from 10^6^ to 10^−2^ TCID_50_ for CVS-11 and from 10^4^ to 10^−2^ TCID_50_ for EBLV-1. The Cq values increased with the viral load. The 95% confidence interval was calculated for all CVS-11 and EBLV-1 tested dilutions (Figure 4(c)).

### 3.7. Matrix Effect

Brain tissue is the preferred specimen for* postmortem* diagnosis in both humans and animals, but this sample is not feasible for* antemortem* diagnosis. In the latter case, diagnosis is based on detection of virus or viral RNA in saliva, neck skin biopsy, or an impression of the cornea. To validate the generic lyssavirus qPCR on other matrices than brain tissue, CSF, saliva, and urine were spiked with different virus doses, ranging from 10^5^ to 10^−1^ TCID_50_ for RABV (CVS-11) and from 10^4^ to 10^−2^ TCID_50_ for EBLV-1. Each sample was analysed in 3 different runs (RNA extraction followed by qPCR) and results were expressed in mean Cq values (Table 5). PCR inhibition and RNA quality were checked by using r18S ribosomal RNA gene amplification. Virus could be detected in all tested matrices (saliva, CSF, and urine). The limit of detection for EBLV-1 was 10^0^ TCID_50_ in CSF, saliva, and urine. The limit of detection for RABV was also 10^0^ TCID_50_ in urine but appeared higher in CSF (10^1^ TCID_50_) and in saliva (10^2^ TCID_50_).

## 4. Discussion

Real-time PCR provides significant methodological benefits for virus detection [34]. RT-PCR offers many advantages for rabies diagnosis, due to its high sensitivity, rapidity, no interference by inhibitors of virus infectivity or antibodies, practicability for samples which may contain only minute amounts of virus, such as cerebrospinal fluid or bat brain, and the possibility to quickly determine the species and molecular phylogeny of the isolate. Several RT-PCR protocols for the detection of rabies virus have been published during the past decade. All protocols that recognise multiple lyssavirus species use degenerate primers [30, 33, 35–39] and most of them use the JW12 primer published by Heaton et al. 1997 [35]. A cocktail of nondegenerate/degenerate primers is only used in the protocols published by Heaton et al. in 1997 [35] and Black et al. in 2003 [36]. Three protocols are designed as a two-step PCR and all of them use a gel-based DNA system detection [30, 35, 39] while others are real-time one-step protocols [33, 36–38]. Only two protocols were validated for 7 species [30, 33]. The RT-PCR of Vázquez-Morón et al. [30] uses a nested classical PCR system with degenerate primers and a gel-based DNA detection system. Real-time RT-PCR is however a more rapid and potentially more sensitive technique [34, 40]. Recently, Hayman et al. [33] validated the use of a real-time RT-PCR protocol for the detection of 7 lyssavirus species. They used a set of mildly degenerate primers that contained one or two degenerate bases per primer and were originally designed for the recognition of RABV, EBLV-1, and -2.

In this study, a two-step generic lyssavirus qRT-PCR, capable of detecting 7 lyssavirus species (RABV, LBV, MOKV, DUVV, EBLV-1, EBLV-2, and ABLV), was developed and validated. The two-step approach allows maximum sensitivity in order to detect virus in typically small-size samples, such as bat brains or samples which contain only minute amounts of virus, such as CSF. A good* in silico* match of the primers with the N gene of the remaining 6 species (WCBV, IRKV, KHUV, IKOV, SHIBV, and BBLV) was also demonstrated. A cocktail of primers with 17 to 30% of degenerate bases, taking into account the variability of the different lyssavirus species, was designed for the assay. The primers target well-conserved regions of the N gene. Indeed, ample sequence data are available for the N gene and most of the published RT-PCR protocols use primers for the N gene [30, 33, 36–39, 41–43]. A large set of samples obtained from wild and domestic animals (bat, red fox, dog, and cat), human patients with nonrabies encephalitis, interlaboratory ring trials, and naturally and experimentally infected animals (bat and mice) was tested. To check for PCR inhibition and RNA quality, the cellular r18S ribosomal RNA gene was used as a target. The r18S ribosomal RNA has been shown to be more reliable than *β*-actin [42, 44]. The diagnostic specificity was 100% and sensitivity proved superior to the fluorescent antigen test. The limit of detection was ≤1 TCID_50_. Sequence analysis of the amplicon unequivocally assigned the correct species. The vesicular stomatitis virus (belonging to the related genus* Vesiculovirus*) was not recognized, confirming the selectivity of the degenerate primers.

This qRT-PCR protocol is now routinely used for rabies surveillance in Belgium. Since 2001, Belgium has been officially declared free of the classic rabies virus. However, bats are an important reservoir of lyssaviruses in Europe [45] and are still collected and tested in the frame of the Belgian rabies surveillance system [46].

In experimentally infected mice, viral RNA was first detected at 3 DPI in the brain, whereas first symptoms appeared much later at 8 DPI. Symptoms involved depression, loss of body weight, ruffled fur, and paralysis of the hind limbs. In general, results of the generic lyssavirus qRT-PCR, RABV-specific qRT-PCR, virus titration, and FAT correlated well and provided similar kinetic profiles throughout the infection. The qRT-PCR proved however to be more sensitive than virus titration and FAT. Indeed, 11 out of 55 mice tested negative by FAT and positive by qRT-PCR. Also, 11 out 55 qRT-PCR-positive mice tested negative by RTCIT. Cycle thresholds obtained by the generic and the RABV-specific qRT-PCR were highly correlated.

The virus species in the positive samples could be identified by sequencing. For some species, we observed different melting temperatures and curves, providing an early indication of the species prior to sequencing. The melting temperature, determined with the CFX96 system from Biorad, was different for RABV, LBV, MOKV, EBLV-1, and EBLV-2, whereas DUVV and ABLV had the same melting temperature (Figure 3). The number of strains tested per species was however insufficient to accurately define the melting curve differentiation. It is also possible to obtain a more precise calculation by high resolution melt analysis [47], which may theoretically allow to discriminate all species prior to sequencing. This was not done for this study.

To further assess the exactitude of our qPCR method, we participated to the consecutive international ring trials organised by ANSES (Nancy, France) from 2009 to 2013. Up to now, we reported very good compliant results in each trial. Moreover, we participated in the Epizone ring trial organised by the Friedrich-Loeffler-Institute (FLI, Germany) in 2011. The panel consisted of 30 samples prepared by the FLI and included RNA of 26 RABV isolated in different countries and years, an EBLV-1 and an EBLV-2 RNA sample, and a log10 serial dilution of RABV. We correctly reported all negative samples and 28 of the 30 positive samples, representing a sensitivity of 93.3%. Two samples with a Cq value >40 were considered as negative samples. These two false negatives could have been due to the fact that the samples were extracted and prepared in RSB50, a buffer different from the one used in our protocol. The RSB50 buffer contains the carrier poly A, which forced us to use a gene-specific reverse transcription kit (qScript Flex cDNA Synthesis kit, Quanta BioSciences, Gaithersburg, USA). Only 3 of the 16 laboratories submitted 100% concordant results for RABV diagnosis [48].

Despite the presence of 17 to 30% of degenerate bases in some of the primers, the lyssavirus qRT-PCR proved to be highly sensitive, specific, and reproducible. In our national reference laboratory, this technique is now used as the method of choice for* antemortem* rabies diagnosis and analysis of small-size samples, such as bat brain.

## Figures and Tables

**Table 1:** FAT and RTCIT-negative samples from various species used for the evaluation of the specificity of the lyssavirus qRT-PCR.

Species	Matrix	Provider	*n*	FAT/RTCIT	Generic lyssavirus qRT-PCR
Bats (*Pipistrellus*, *Myotis,* and *Eptesicus serotinus*)^1^	Brain tissue	Rabies NRC, WIV-ISP, Belgium	100	Negative	Negative
Red Fox (*Vulpes vulpes*)^1^	Brain tissue	Rabies NRC, WIV-ISP, Belgium	10	Negative	Negative
Dog (*Canis Lupus familiaris*)^1^	Brain tissue	Rabies NRC, WIV-ISP, Belgium	10	Negative	Negative
Cat (*Felix cati*)^1^	Brain tissue	Rabies NRC, WIV-ISP, Belgium	10	Negative	Negative
Mouse (*Mus Musculus*)^2^	Brain tissue	Rabies NRC, WIV-ISP, Belgium	20	Negative	Negative
Human (*Homo Sapiens*)^3^	Cerebrospinal fluid	Rabies NRC, WIV-ISP, Belgium	10	Negative	Negative
Human (*Homo Sapiens*)^3^	Saliva	Rabies NRC, WIV-ISP, Belgium	5	Negative	Negative
Human (*Homo Sapiens*)^3^	Skin tissue	Rabies NRC, WIV-ISP, Belgium	1	Negative	Negative

^1^Samples collected on the Belgian territory between 2007 and 2012 in the frame of the national surveillance system to guarantee the rabies-free status of Belgium.

^2^Specific-pathogen-free female Swiss outbred laboratory mice obtained from Harlan (Boxmeer, The Netherlands).

^3^Patients with encephalitis symptoms sent to the national reference centre for rabies virus, Belgium.

**Table 2:** Rabies virus-positive samples used to assess the diagnostic sensitivity of the lyssavirus qRT-PCR.

Positive samples	Provider	Strain reference	Matrix	*n*
Classical rabies virus RABV	ANSES, France^1^	Ariana, Tunisia	Dog brain homogenate	3
	ANSES, France^1^	CVS-27	Mouse brain homogenate	3
	ANSES, France^1^	GS7, France	Fox brain homogenate	7
	ANSES, France^1^	Raccoon, Poland	Raccoon dog brain homogenate	1
	ANSES, France^1^	201020958, Spain	Mouse brain homogenate	2
	ANSES, France^1^	Cn Viv Estonie 10–12, Estonia	Mouse brain homogenate	1
	Rabies NRC, ISP-WIV	Strain fox, Belgium (1995)	Red fox brain homogenate	1
	Rabies NRC, ISP-WIV	Strain fox, Luxembourg (2000)	Pony brain homogenate	1
	Rabies NRC, ISP-WIV	CB-1, Maroc (2007)	Dog brain homogenate	1
	ATCC, USA	CVS-11 (VR959)	Mouse brain homogenate (experimentally infected mice at Rabies NRC, WIV-ISP, Belgium)	20

Lagos bat virus LBV	Centre des Ressources Biologiques, Pasteur Paris Institute, France	CRBIP8.14	Cell culture lysate	1

Mokola virus MOKV	Centre des Ressources Biologiques, Pasteur Paris Institute, France	CRBIP8.27	Cell culture lysate	1

Duvenhage virus DUVV	ANSES, France^1^	96132, SA (fixed strain)	Mouse brain homogenate	1
	Centre des Ressources Biologiques, Pasteur Paris Institute, France	CRBIP8.28	Cell culture lysate	1

European bat lyssavirus-1 EBLV-1	ANSES, France^1^	EBLV-1a, France	Mouse brain homogenate	3
	ANSES, France^1^	EBLV-1b, France	Mouse brain homogenate	4
	WIV-ISP, Belgium^2^	AF-2010, Spain	Bat brain homogenate (Naturally infected *Eptesicus serotinus* bat)	1
	ANSES, France^1^	R75, Spain	Mouse brain homogenate	1
	Pasteur Paris Institute, France^3^	8919FRA, France (isolated from *Eptesicus serotinus* bat)	Mouse brain homogenate (experimentally infected mice at Rabies NRC, WIV-ISP, Belgium)	5

European bat lyssavirus-2 EBLV-2	ANSES, France^1^	EBLV-2 VLA P3, United Kingdom	Mouse brain homogenate	1
	ANSES, France^1^	EBL2 RV1787, United Kingdom	Mouse brain homogenate	1
	ANSES, France^1^	EBLV-2, United Kingdom	Mouse brain homogenate	5

Australian bat lyssavirus ABLV	ANSES, France^1^	ABLV, Australia	Mouse brain homogenate	4

^1^Samples obtained through participation to consecutive an interlaboratory proficiency tests organised by the European Union reference laboratory of rabies (ANSES, Nancy, France) between 2009 and 2013. Samples were reconstituted in 1 mL sterile, nuclease-free, and distilled water (Robardet et al., 2011 [26]).

^2^Van Gucht et al., 2013 [27].

^3^Bourhy et al., 1992 [28].

**Table:** (a)

Rabies virus	Identification	GenBank accession number
RABV	CVS-11	GQ918139
RABV	9174GSFRA	U22474
RABV	9107MAR	U22852
LBV	LBVSA2006	EF547452
LBV		U22842
MOKV		NC006429
MOKV		U22843
DUVV		U22848
DUVV		EU623438
EBLV-1	891FRA	U22845
EBLV-1	8615POL	U22844
EBLV-2	9007FIN	U22846
EBLV-2	9018HOL	U22847
ABLV		AF006497
ARAV		EF614259
ARAV		AB094438
KHUV		EF614261
IRKV		EF614260
IRKV	THChina12	JX442979
WCBV		EF614258
SHV		GU170201
BOKV	21961	JF311903
IKOV	Isolate RV2508	JX193798
IKOV		NC018629

**Table:** (b)

Name	Tm	Sequence 5′-3′	Position (for RABV-CVS11)	Use of primers
RAB PCR1 F	60,2°C	AYAARATGTGYGCIAAYTGGAGYA	572–595	Generic lyssavirus PCR1
RAB PCR1 R	61,8°C	ACIGCRTTSGANGARTAAGGAGA	892–914	Generic lyssavirus PCR1
RAB qPCR F	62,1°C	GTIGGVACDGTIGTIACHGCHTA	676–698	Generic lyssavirus qRT-PCR
GRAB2R	61°C	TCYTGHCCIGGCTCRAACAT	814–833	Generic lyssavirus qRT-PCR
RAB CVS11 F	68,1°C	GTGGGCACAGTCGTCACCGCTTA	676–698	RABV-specific qRT-PCR
RAB CVS11 R	60,85°C	TCTTGCCCTGGCTCGAACAT	814–833	RABV-specific qRT-PCR
VETINHF2	60,8°C	GTTGATTAAGTCCCTGCCCTTT	/	r18S qPCR
VETINHR1	60,8°C	GATAGTCAAGTTCGACCGTCTT	/	r18S qPCR

The r18S ribosomal RNA gene primers were designed to amplify RNA from multiple species.

**Table 4:** Comparison of the sensitivity between the two-step lyssavirus qRT-PCR and the one-step qRT-PCR published by Hayman et al. in 2011 [33].

Dilutions	RABV strain		EBLV-1 strain	
	Two-step developed assay	One-step published assay	Two-step developed assay	One-step published assay
	Cq value	Cq value	Cq value	Cq value
1.00*E* + 00	9.52	17.41	6.77	19.51
1.00*E* − 02	12.58	23.80	11.40	26.92
1.00*E* − 03	17.59	27.31	17.07	30.70
1.00*E* − 04	21.97	30.95	21.37	33.70
1.00*E* − 05	26.16	34.29	24.98	37.36
1.00*E* − 06	**30.94**	**38.10**	**28.35**	**ND**
1.00*E* − 07	**34.30**	**ND**	**ND**	**ND**

RNA was extracted from infected cell culture supernatants, serially diluted and used to generate cDNA. The cDNA samples were tested by both qRT-PCR protocols. ND: no signal detected.

**Table:** (a)

RABV (TCID_50_)	CSF	Saliva	Urine
1.00*E* + 05	19.65	18.80	18.53
1.00*E* + 04	24.50	23.39	23.13
1.00*E* + 03	28.12	27.17	26.85
1.00*E* + 02	33.15	32.75	30.56
1.00*E* + 01	40.53	ND	39.67
1.00*E* + 00	43.54	ND	39.78
1.00*E* − 01	ND	ND	ND
1.00*E* − 02	ND	ND	ND

**Table:** (b)

EBLV-1 (TCID_50_)	CSF	Saliva	Urine

1.00*E* + 04	14.56	15.31	14.50
1.00*E* + 03	18.11	18.34	18.84
1.00*E* + 02	22.72	23.79	22.17
1.00*E* + 01	27.74	28.90	28.06
1.00*E* + 00	33.10	28.17	28.67
1.00*E* − 01	ND	ND	ND
1.00*E* − 02	ND	ND	ND

Saliva, CSF, and urine samples were spiked with decreasing doses of RABV or EBLV-1. Three independent runs with each time two repeats were performed per virus dose (10-fold serial dilution). The limit of detection for RABV was 10^0^ TCID_50_ in urine, 10^1^ TCID_50_ in CSF, and 10^2^ TCID^50^ in saliva. For EBLV-1, the limit of detection was 10^0^ TCID_50_ in CSF, saliva, and urine. ND: signal not detected.
